# Asymmetric developmental change regarding the effect of reward and punishment on response inhibition

**DOI:** 10.1038/s41598-019-49037-9

**Published:** 2019-09-09

**Authors:** Mami Miyasaka, Michio Nomura

**Affiliations:** 1 Department of Cognitive Psychology in Education, Graduate School of Education, Kyoto University, Kyoto, Japan; 2 Japan Society for the Promotion of Science, Tokyo, Japan; 3 Present Address: Department of Human Development, International Pacific University Women’s College, Ehime, Japan

**Keywords:** Motivation, Social behaviour, Human behaviour

## Abstract

Reward and punishment influence inhibitory performance, but developmental changes in these effects are not well understood. Our aim was to understand the effects of potential reward gains and losses (as indices of reward and punishment) on response inhibition among children and adolescents. We conducted financial and non-financial go/no-go tasks with 40 boys (8- to 15-year-olds). Participants gained or lost money depending on their performance on the financial task, and score rankings were compared to participants on the non-financial task. We found that adolescents’ inhibitory control, as reflected in their reaction times when they made inhibitory errors, was lower in the reward-present condition than in the reward-absent condition, although accuracy was higher when the reward was available for all participants. Additionally, inhibitory control, specifically among adolescents, was higher for financial feedback than for non-financial feedback. These results suggest that the effects of reward and feedback type on motor impulsivity differ as a function of developmental stage. We discuss the theoretical implications of the present findings in terms of the interaction between emotional feedback and response inhibition among children and adolescents.

## Introduction

Inhibitory control allows humans to refrain from processing unnecessary stimuli and behave appropriately in various situations^1^. Response inhibition is a particularly important type of inhibitory control, which involves suppression of proponent or automatic responses^2,3^. Deficits in response inhibition can lead to detrimental impulsivity in daily life^4^.

Actions are carried out through interactions between the senses, perception of information, and higher-order cognition in response to environmental input (i.e. a stimulus). Emotions can influence cognition^5^ during this process, as can feedback (e.g. rewards for success and punishment for errors). Feedback could have particular effects on cognitive control, including inhibitory control^6,7^. However, feedback (such as financial incentives) does not necessarily improve task performance^8^. For instance, Bonner *et al*. (2000) found that whether financial incentives have a positive effect on task performance is significantly related to the type of task and incentive scheme. This was based on a review of 131 published laboratory experiments that examined the effects of financial incentives on task performance. These observations suggest that multiple factors should be considered when discussing the effect of incentives on task performance.

The adolescent period has been a key research focus due to the dynamic biological and psychological changes occurring during this time^9^. Furthermore, knowledge regarding maturation—namely when a person becomes an adult—contributes to law and policy^10,11^. Although goal-directed behaviour is known to mature throughout adolescence^12^, reward sensitivity (which underlies how rewards affect behaviour—such as risky decisions) appears to develop in a specific manner, with adolescents presenting with specific responses for incentives in comparison to other age groups^13–16^.

The current study also focused on the difference between middle-school-age (8 to 12-years-old) and early adolescent children^17^. Although the period of adolescence is difficult to define, as neurobiological maturation is not uniform across the brain^11^, we defined early adolescence as 13 to 17-years-old, following Newman and Newman (1976) and other previous studies^11^; we particularly focused on people under the age of 15.

Prior fMRI studies have shown limitations in adolescents’ reward assessment and heightened reactivity to reward anticipation as compared to adults^13,14^. Although ventral striatal activation was greater for adolescents in comparison to adults during reward receipt^14,15,18^, both groups demonstrated greater activation during winning than when failing to win^14^; results from other studies have been mixed, with low activation among adolescents during reward anticipation^19^. Hence, the effect of incentives on inhibitory control might not necessarily be in the positive direction.

Recently, developmental changes with regard to the interaction between incentives and higher cognitive processes, such as inhibitory control, have also been researched. A prior study suggested that using value signals for cognitive control continues to mature through adolescence, even though young children can detect value in their environment and use the information for value-guided cognitive control^20^. In spite of the adolescents’ specific response pattern in the incentive-processing brain region, the enhancing effect of incentives during inhibitory control among adolescents has been observed^18,21,22^. However, diminished performance on inhibitory tasks within incentivised conditions among adolescents has also been revealed. Based on results from recent fMRI studies^18^, the influence of incentives on adolescents’ cognitive performance seems to differ as a function of incentive-related phase: anticipation, response preparation, and receipt. Geier and Luna (2012) compared the effect of incentives (i.e. reward, punishment, neutral) on inhibitory control among 13- to 15-year-old adolescents, 15- to 17-year-old adolescents, and 18- to 29-year-old adults using an oculomotor paradigm. In a comparison of error rates across incentive types, they observed more inhibitory errors during reward as compared to loss trials, and this effect stemmed from high error rates for reward trials and low error rates for loss trials; this was observed among 13- to 15-year-old younger adolescents when performance was collapsed across age groups. In terms of latencies, they observed that reward and loss latencies decreased relative to neutral latencies for younger adolescents. These results seemed to reflect the idea that incentives have a greater effect on voluntary responses for younger adolescents^23^. Thus, younger adolescents may have difficulty controlling motor inhibition in the presence of reward. Relatedly, Padmanabhan, Geier, Ordaz, Teslovich, and Luna (2011) investigated developmental trends in the interaction between financial motivation (i.e. reward gain) and inhibitory control among children, adolescents, and adults using another oculomotor paradigm. The authors revealed that immaturity in orbital frontal cortex development, which might support executive processing of rewards, and enhanced activation in the ventral striatum among adolescents, might reflect reward-based decision-making in this age group. Although these behavioural results suggest improved inhibitory performance, it might be the case that incentives influence adolescents to be more impulsive with other types of incentive types and/or during types of inhibition tasks, especially for motor inhibition tasks that require both ‘go’ and ‘stop’ responses.

Interestingly, Geier and Luna (2012) also found that 13- to 15-year-olds performed as well as 18- to 29-year-olds during financial punishment trials (i.e. reward loss). In addition, Paulsen, Hallquist, Geier, and Luna (2015)^24^, who utilized a longitudinal incentivised oculomotor paradigm, also showed that the performance on reward trials was more stable than on loss trials within individuals. This different response for reward and punishment in adolescence could be explained by a stronger reward-related system supported by the ventral striatum and a weaker-harm-avoidant system supported by the amygdala and/or poor regulatory control related to the prefrontal cortex^25^.

Another important concept is social re-orientation in adolescence. This occurs in connection with alterations in neuronal processes that are brought about by hormonal restructuring, maturation, and learning, which likely lead to changes in the processing of social stimuli and/or behaviour in a social context^9^. Relatedly, Blakemore and Mills (2014) suggested the importance of social context in studies on adolescents. For instance, adolescents appear to be more sensitive to their social environment than are boys/girls <10 and ≥20-years-old. This may mean that the effects of non-financial feedback on inhibitory performance differ between adolescents and older children. Non-financial feedback, such as praise and blame, is also a familiar form of reward/punishment in daily life. Recently, several studies have addressed differing effects of financial and non-financial feedback^26,27^. For example, Kohls *et al*. (2009) assessed children both with and without attention-deficit hyperactivity disorder (ADHD) and observed that both financial and non-financial rewards improved inhibitory control for both groups. Non-financial rewards, however, were less effective for children without ADHD than for those with ADHD. The contrasting effects of these forms of feedback on inhibitory control, and their developmental changes, should be clarified among individuals without developmental disorders, as well as in clinical populations. This should further our understanding on this important topic. Another type of emotional go/no-go task using appetitive (happy faces) and neutral cues (calm faces)^28^, which helps assess stimulus-driven information processing^29^, also produces specific reactions as a function of social stimulus. For instance, Somerville *et al*. (2011) observed that appetitive cues led to enhanced ventral striatum activation and reduced inhibitory performance among adolescents. These observations highlight adolescents’ sensitivity for socially appetitive cues.

The aim of the present study was to investigate the effect of financial/non-financial reward and punishment on response inhibition and developmental differences between children and adolescents. To this end, and in order to expand our theoretical understanding of certain clinical interventions (e.g. parental training or social skills training), we conducted go/no-go tasks under financial and non-financial feedback conditions with 8- to 15-year-old boys. This age range was chosen to include participants likely to be most influenced by punishment when performing inhibitory control tasks (i.e. 13- to 15-year-olds)^23^ as well as individuals (i.e. 8- to 12-year-olds) for whom the effect might be lower. Participants gained or lost money depending on their performance on the financial task, and their score ranking was compared to other participants within the non-financial task. We used rankings in relation to other participants as non-financial feedback because this is a major form of feedback in educational settings^30^. Of course, prudence must be exercised in the treatment of rankings because such scores can either facilitate frustration or motivation for different children. As several research groups have pointed out, many studies assessing children and adolescents arbitrarily separate participants by age and compare cognitive characteristics for each developmental stage^31^. For this reason, the age range often differs between studies and might obscure a theoretical understanding of this topic. Hence, we statistically analyzed the effect of age as a continuous variable.

Our hypotheses were as follows: With adolescents typically demonstrating high reward sensitivity^22,23,28^, we assumed that the adolescent group would display less inhibitory control in the reward-present condition than in the reward-absent condition; the same was not expected for the middle school age group. Similarly, if the effect of punishment on inhibitory control changes between childhood and adolescence, we would expect to see age-dependent differences within the punishment condition. As adolescents are also sensitive to social context^9,31^, we expected that a non-financial reward/punishment would lead to greater impulsivity for adolescents than would a financial reward/punishment. On the other hand, this difference might not be observed among the child group, as children do not present this sensitivity as much as adolescents do. Hence, we exploratorily compared the effects in adolescents to those in children.

## Results

Descriptive statistics are presented in Table S1. We did not find age-related differences in participants’ profiles within a regression analysis with age as a predictor variable (ADHD-RS-IV: *β* = −1.826, *SE* = 1.977, 95% confidence interval [CI] = −5.826–2.174, *p* = 0.361; ASSQ: *β* = −0.150, *SE* = 0.409, 95% CI = −0.977–0.677, *p* = 0.715).

### Commission Error RT (CERT)

When comparing model fit, the Akaike information criterion [AIC] of the linear model and quadratic model did not differ (*p* = 0.495). The AIC of the linear model was 3020.8 and 3022.3 for the quadratic model. As the AIC of the former model was lower, we applied the linear model for the following analysis.

**Figure 1:**
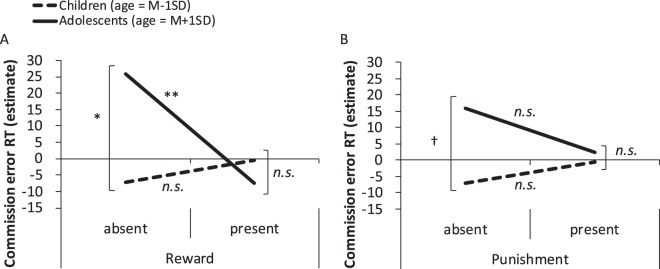
Results of multiple regression analyses with a mixed effect model for the CERT. Higher values on the vertical axis indicate better inhibitory performance (i.e. less impulsivity). (**A**) Shows the relationships between the presence and absence of reward and age. (**B**) Shows the relationships between the presence and absence of punishment and age.

**Figure 2:**
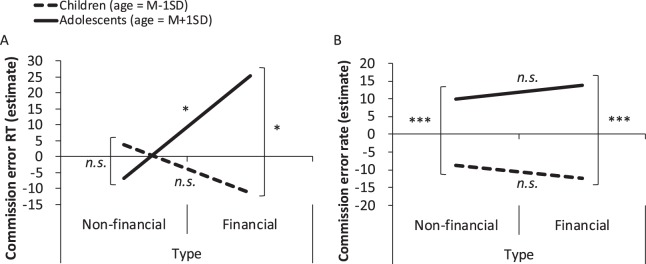
Results of multiple regression analyses with a mixed effect model for the CERT and CER. The relationship is between feedback type and age. Larger values in both (**A**,**B**) indicate better inhibitory control, as we inverted the valence of the values of the vertical axis in (**B**).

According to results of the multiple regression analysis (AIC = 2936.678, *f*^2^ = 0.130, power (1-*β*) = 0.186; see Table S2), the main effect of reward was marginally significant (*β* = −13.346, *SE* = 7.430, 95% CI = −27.861–1.157, *p* = 0.080), suggesting that reward CERT in the reward-present conditions were marginally shorter than in the reward-absent conditions. We did not observe any other main effects (i.e. age, punishment, and feedback type). The interaction between age and reward (*β* = −8.830, *SE* = 3.420, 95% CI = −15.585–2.240, *p* = 0.013; Fig. 1A) and age and feedback type (*β* = 10.426, *SE* = 4.563, 95% CI = 1.757–19.625, *p* = 0.028; Fig. 2A) were significant.

Further analyses for the interaction between age and reward revealed that the effect of reward was only significant for adolescents (M_Age_ + 1 SD [i.e. 13.81 years old]: *β* = −33.302, *SE* = 10.600, *p* = 0.003; M_Age_ − 1 SD [i.e. 9.26 years old]: *β* = 6.610, *SE* = 10.840, *p* = 0.546), with the CERT being shorter in the reward-present than in the reward-absent condition. Furthermore, the effect of age was only observed within the reward-absent condition (present: *β* = −1.613, *SE* = 2.224, *p* = 0.473; absent: *β* = 7.656, *SE* = 3.190, *p* = 0.022), with the CERT being shorter among children than among adolescents (see also Fig. 1A). Additionally, we did not observe a significant interaction between age and punishment (*β* = −4.368, *SE* = 3.033, 95% CI = −10.265–1.505, *p* = 0.155; Fig. 1B).

Regarding the interaction between age and feedback type, the effect of feedback type was only significant among adolescents (M_Age_ + 1 SD: *β* = 32.203, *SE* = 13.792, *p* = 0.025; M_Age_ − 1 SD: *β* = −14.921, *SE* = 14.217, *p* = 0.302), with the CERT being shorter in the non-financial than in the financial condition. The effect of age was significant within the financial condition (financial: *β* = 7.239, *SE* = 3.414, *p* = 0.041; non-financial: *β* = −1.934, *SE* = 2.840, *p* = 0.500), with the CERT being shorter among children than among adolescents (see also Fig. 2A).

### Commission Error Rate (CER)

There was no difference in model fit between the linear and quadratic model (*p* = 0.689). The AIC of the linear model was 2624.0 and 2625.8 for the quadratic model; thus, we applied the linear model for the following analysis.

According to the multiple regression analysis (AIC = 2599.249, *f*^2^ = 0.474, power (1-*β*) = 0.921; see Table S3), the main effects of age (*β* = −4.968, *SE* = 0.813, 95% CI = −6.523–3.416, *p* < 0.001) and reward (*β* = −3.191, *SE* = 1.318, 95% CI = −5.726–0.655, *p* = 0.017) were significant. The effect of punishment was not significant (*β* = −2.222, *SE* = 1.594, 95% CI = −5.339–0.896, *p* = 0.171). Specifically, adolescents exhibited a lower CER, and all participants showed a lower CER in the reward-present condition. The interaction between age and feedback type was marginally significant (*β* = −1.681, *SE* = 0.876, 95% CI = −3.399–0.010, *p* = 0.062; Fig. 2B). Further analyses revealed that the effect of feedback type was not significant for either group (M_Age_ + 1 SD: *β* = −3.960, *SE* = 2.764, *p* = 0.159; M_Age_−1SD: *β* = 3.639, *SE* = 2.643, *p* = 0.177). However, the direction of the *β* was opposite between the two groups, namely that the CER was lower in the financial than in the non-financial condition for adolescents and lower in the non-financial than in the financial condition for children. A significant main effect of age was observed for both types of feedback (financial: *β* = −5.835, *SE* = 1.043, *p* < 0.001; non-financial: *β* = −4.198, *SE* = 0.870, *p* < 0.001). The CER was lower among adolescents than among children in no-go tasks.

## Discussion

We investigated the interaction between different feedback types (financial and non-financial reward and punishment) on response inhibition as a function of developmental stage (children and adolescents). The main effect of reward was significant for the CER, and fewer errors were made when rewards were available.

Given the significant main effect of reward on the CER, rewards appear to facilitate accurate performance. This is consistent with some previous studies observing the enhancing effect of incentives on inhibitory performance^18,21,22^. However, the downside of this occurs when we focus on reaction times for commission errors.

We did reveal a significant interaction between age and reward for the CERT: in the reward-present condition, the CERT was shorter than in the reward-absent condition among adolescents (*M*_age_ + 1 SD) but not children (*M*_age_ − 1 SD). This suggests that inhibition of ongoing motor responses is disturbed in the presence of a financial or non-financial reward in adolescence. Furthermore, we found a significant interaction between age and feedback type for the CERT, indicating that the inhibitory control was high for financial feedback among adolescents.

Findings regarding the interaction between age and reward corresponds to Van Leijenhorst *et al*. (2010), who explained this phenomenon as resulting from changes in reward sensitivity based on neural development from childhood to adolescence. Several fMRI studies also support these findings, wherein activation in the ventral striatum in response to uncertain rewards does not necessarily associate adolescents’ task performance^22,28^. Thus, rewards have a strong effect on reflective response inhibition, as well as higher-order cognitive functions (e.g. decision-making). These observations suggest that implementing rewards to improve response inhibition might be inadvisable for adolescents, as adolescents may be inclined to increase impulsive behaviours.

We hypothesized that age-dependent differences in the punishment condition would emerge if the effect of punishment on inhibitory control changes between childhood and adolescence. In brief, we estimated that the effect of punishment would only be observed in the adolescent group. Unexpectedly, the punishment by age interaction was not significant. In the current study, neither improvement nor impediment in response inhibition due to punishment emerged in the adolescent group. Based on this differential effect of reward and punishment, we speculate that the role of punishment on inhibitory control is similar from childhood to adolescence, while reward produces specific effects in adolescence. Palminteri, Kilford, Coricelli, and Blakemore (2016)^32^ demonstrated that adolescents learn from reward and punishment in an asymmetrical way, whereby punishment is less effective than reward. As adolescents pursue reward, and do not consider punishment to the same extent, our adolescent participants might not have engaged in impulsive responses in order to avoid punishment (i.e. less inhibition) in the punishment condition, unlike what was observed in the reward condition.

According to the dual competition model, emotional content (e.g. fearful faces) enhances sensory processing of emotional stimuli^29,33^; our go and no-go stimuli were neutral, as they were just simple figures. Hence, when participants’ inhibitory performance changed with the reward/punishment condition, it is likely that improvement occurred due to feedback incentives rather than the stimuli themselves. This effect is considered state-dependent, such that executive control improves because affective content enhances sensory representations of emotional items and the motivation to allocate resources so as to maximize potential rewards^29^. Our results suggest that older children and adolescents use reward information, rather than punishment, to decide how to allocate their cognitive resources. From there, reward information leads to impediments rather than improvements to response inhibition in some cases, especially for adolescents. Similarly, our results also suggest that the usefulness of certain feedback types (i.e. financial or non-financial) changes with age.

The present study has a few limitations. First, the sample size was small; in actuality, the power (1-*β*) of CERT was too small. We determined that the requisite sample size was 34, similar to our actual sample size, when we calculated the a priori sample needed to investigate reward by age interactions using G*Power 3.1^34,35^ with the following parameters: (i) medium effect size based on high reward sensitivity^16^ and changes to cognitive performance and brain activity through incentives^22^ and (ii) a power of 0.8. It is possible that our sample size was not sufficiently large, considering that we did not conduct any power analysis for other hypothesized interactions. The reason for this was that we could not find available behavioural data from past research to use as a model case. Nevertheless, our results are still congruent with previous studies. Furthermore, our corresponding effect size also exceeded Cohen’s (1988)^36^ the criteria for small (i.e. CERT) and large (i.e. CER) effects. Additionally, our results contribute to theory building in this research area. Second, although we set the number of trials based on those of a previous study, the participants of Demurie *et al*. (2016)^37^ included not only typically developing children but also children with developmental disorders who usually demonstrate high inhibitory errors; the number of no-go trials seems relatively small for detecting sufficient commission errors. Hence, future studies should include more no-go trials, especially if they target typically developing adolescents. Finally, the study design was cross-sectional; thus, in order to better evaluate any developmental effects, a future longitudinal study would be ideal. Thirdly, our participants were all boys; whether our results generalize to other groups (i.e. girls) should be addressed in the future. Finally, as we did not include adults, we cannot discuss whether the effect of reward/punishment on adolescents’ inhibitory control is the same or different from what is observed in adulthood. Thus, future work should address a more expansive developmental range, from childhood into adulthood.

## Method

### Participants

Forty-six boys were recruited through research agencies (Fieldwork by Rakuten Research, Inc., Japan and CROèe Inc., Japan) and flyers. We chose this sample size based on previous research^16^ observing interactions between age and reward sensitivity, as reward sensitivity should help reflect interactions between age and incentivised behavioural performance. Eight- to 15-year-old children were recruited, as the age cut-off for compulsory education is 15 in Japan: the range for elementary school is 6 to 12-years-old and 12 to 15-years-old for junior high school. We recruited only boys because past studies assessing brain activity during inhibitory tasks have revealed sex differences^38,39^. Our main exclusion criterion was the presence of a diagnosed developmental disorder; this is because prior work has shown that individuals with developmental disorders, such as ADHD or autism spectrum disorders, experience high levels of impulsivity^40^, which could be a key confound in our results. We excluded 6 participants: 5 for having a suspected developmental disorder (verified via parental report) and one for not completing the study. Subsequently, we included 40 boys (*M*_age_ = 11.55 ± 2.26; range = 8–15 years old, *M*_ADHD-RS-IV_ = 37.35 ± 27.77, *M*_ASSQ_ = 5.75 ± 5.77) in our analysis. This sample included 23 elementary school students (*M*_age_ = 9.82 ± 1.02) and 17 junior high school students (*M*_age_ = 13.88 ± 0.99). Children/adolescents (hereafter, referred simply as ‘children’) and parents/guardians were instructed to visit the lab twice and were given a 1,000-yen prepaid card for purchasing books after each day as compensation. We obtained consent from both children and parents. All patients signed the institutional review board–approved informed consent prior to the beginning of the experiment. This study was approved by the institutional review board at Kyoto University and was carried out in accordance with approved ethical guidelines.

### Behavioural Measurements

#### Procedure

Financial and non-financial go/no-go tasks were computer-based and programmed in Super Lab 4 (Cedrus Corporation, USA). Programs were presented on a 13-inch computer monitor. The distance from the centre of the display to participants’ eyes was approximately 60 cm. The financial and non-financial tasks were conducted on different days to prevent fatigue and minimize potential training effects. The mean time between sessions was 17.9 ± 5.1 days. To prevent children from counting the money, we used a point system and gave the points gained for each trial, not the total accumulated, as feedback. There were 4 reward–punishment conditions for both tasks. The order of tasks was counterbalanced between participants.

On the first day, children were told the following: (1) You will play a game today and on another day; (2) there are two games in total; (3) it will take approximately 20 minutes to complete one game; and (4) you will be given a prepaid card that you can use to buy books or cartoons after each game day in appreciation for your participation. The instructions for the financial task were as follows: ‘In these games, your points will be increased and decreased. You will get additional cards if you get many points, but your cards will decrease if you get fewer points’. The instructions for the non-financial task were as follows: ‘In this game, your points will be increased and decreased. You will go up in the rankings if you get many points, but you will go down if you get fewer points’.

Each participant received feedback regarding the financial and non-financial tasks after completing each. Every child received an additional 500 or 1,000-yen pre-paid card. We created a point-ranking table for the non-financial task before the experiment and ranked participants according to their points.

#### Go/no-go task

**Figure 3:**
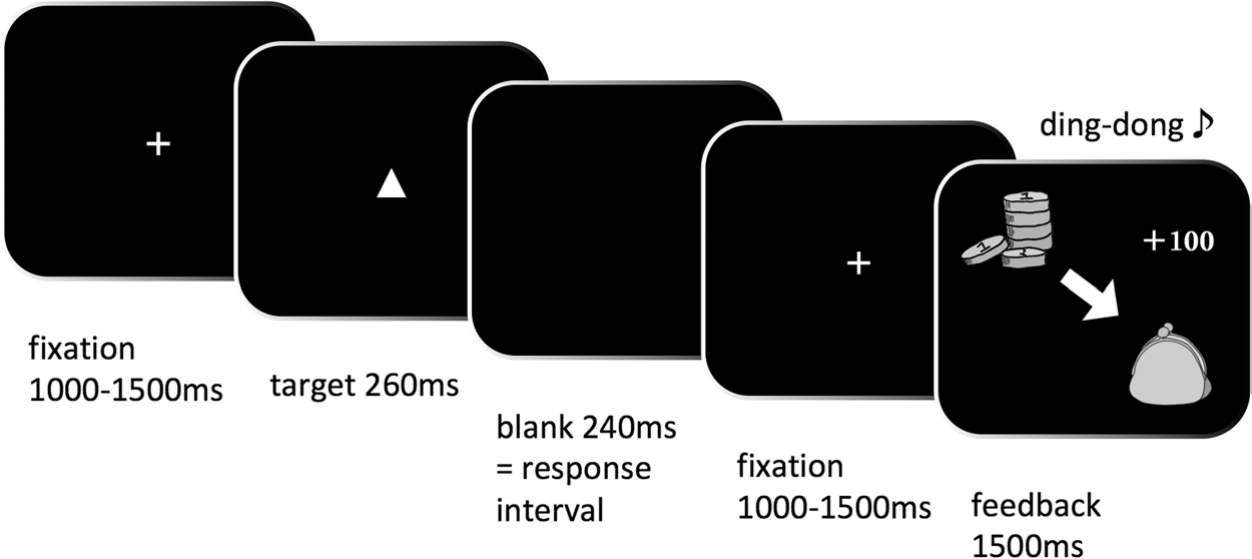
An example of a correct go response during the financial Go/No-go task. In this example, participants were required to push the button.

The go/no-go task measures motor response inhibition, a major form of inhibitory control (Figs 3, S1). We modified Demurie *et al*.’s (2016) version of the task for the present study. The stimuli were a white triangle (active stimuli) or square (passive stimuli). After the first fixation, a target stimulus was presented at the centre of a black screen for 260 ms, followed by a 240-ms blank screen. Participants could respond from the moment of target presentation until presentation of the next fixation (i.e. 500 ms). After the second fixation, a feedback illustration and sound were presented for 1.5 s. The fixations before the target and feedback were presented at random intervals between 1 and 1.5 s. Participants were instructed to push the button (i.e. go response) using the index finger of their dominant hand when active stimuli (i.e. go stimuli) appeared, as quickly and as accurately as possible. Participants were asked to never press the button (i.e. no-go response) when passive stimuli (i.e. no-go stimuli) appeared. The commission error rate, which is a go response for passive stimuli, and the mean reaction time during commission errors were used to measure inhibitory responses.

Before the test stage, participants practiced the task. In the first stage, active and passive stimuli were presented for 1 s, followed by a 3-s blank screen. Three active stimuli and one passive stimulus were presented. The time pressure in the second practice stage was the same as in the test stage but comprised 9 active and 3 passive stimuli. Feedback comprised only whether the participant was successful (correct mark and sound) or failed (error mark and sound).

In the test stage, one block included 24 go trials (75%) and 8 no-go trials (25%), randomly ordered. Participants completed 4 blocks (i.e. 4 reward–punishment conditions), the order of which was counterbalanced between participants. The participants could rest between the blocks.

#### Reward–punishment conditions

We controlled the presence of reward (i.e. reward-absent [RewA], reward-present [RewP]) and punishment (i.e. punishment-absent [PunA], punishment-present [PunP]). Then, we set 4 conditions. In the no reward-no punishment condition (RewA-PunA), points did not change regardless of correct responses (‘go’ for active stimuli and ‘no-go’ for passive stimuli) or incorrect responses (‘no-go’ for active stimuli and ‘go’ for passive stimuli). In the reward condition (RewP-PunA), participants gained 100 points for a correct response, and the points did not change for an incorrect response; conversely, in the punishment condition (RewA-PunP), the points did not change for a correct response, and participants lost 100 points for an incorrect response. Finally, in the reward-punishment condition (RewP-PunP), 100 points were given for a correct response, and 100 points were taken away for an incorrect response.

#### Questionnaires

A parent answered the Japanese version of the ADHD Rating Scale-IV Home Version (ADHD-RS-IV)^41^ to measure the frequency of children’s ADHD-related behaviour, and the Autism Spectrum Screening Questionnaire (ASSQ)^42,43^, which is widely used for identifying autism spectrum disorders. The ADHD-RS-IV index is a percentile score converted from a raw score based on a participant’s age and gender. The ASSQ score range is 0 to 54. We collected these data in order to control for their influence on our main dependent measures.

### Data analysis

All statistical analyses were performed using R 3.3.1 for Mac OS X^44^. Multiple regression analyses with mixed effects models were performed to validate the main effects of age, feedback type (financial or non-financial), existence of reward, and existence of punishment on inhibition and all possible interactions as fixed effects. We coded the financial feedback as 0.5 and the non-financial feedback as −0.5; the reward-present condition was 0.5 and reward-absent condition was −0.5; and the punishment-present condition was 0.5 and punishment-absent condition was −0.5. The fixed effects also included the block order, ADHD-RS-IV score, and ASSQ score as covariates to avoid type 1 errors among target variables. Random effects included intercepts for participants, as well as participant slopes for feedback type, reward, and punishment. The dependent variables were the commission error rate (CER, which is a go response for passive stimuli) and the *M*_RT_ during a commission error (CERT); less inhibitory control was indicated by a high CER and short CERT, reflecting a response that was too incorrect and fast^27,45^. Eighteen participants who did not present any incorrect responses in at least one block were excluded from analysis for CERT (Table S4). Simple slope analyses were conducted for significant interactions. We statistically analyzed the effect of age as a continuous variable and separated participants using a ± 1 SD from the mean age as a criterion only when age-related interactions were found. Furthermore, as age-related changes could produce quadratic associations, we tested both linear association and quadratic association models before conducting our main analysis, with reference to Paulsen *et al*. (2015)^24^.

## Supplementary information

Supplemental information

## Data Availability

The datasets generated and/or analysed during the current study are available from the corresponding author upon request.
