# Supplementary material for: Asymmetric developmental change regarding the effect of reward and punishment on response inhibition
Source: Sci Rep. 2019 Sep 9;9:12882. doi: 10.1038/s41598-019-49037-9 (PMC6734010; doi:10.1038/s41598-019-49037-9)
Supplement: Supplementary file 1 — Supplemental information [file 41598_2019_49037_MOESM1_ESM.pdf]

Miyasaka, M. and Nomura, M. Asymmetric developmental change regarding the effect of reward and punishment on response inhibition.

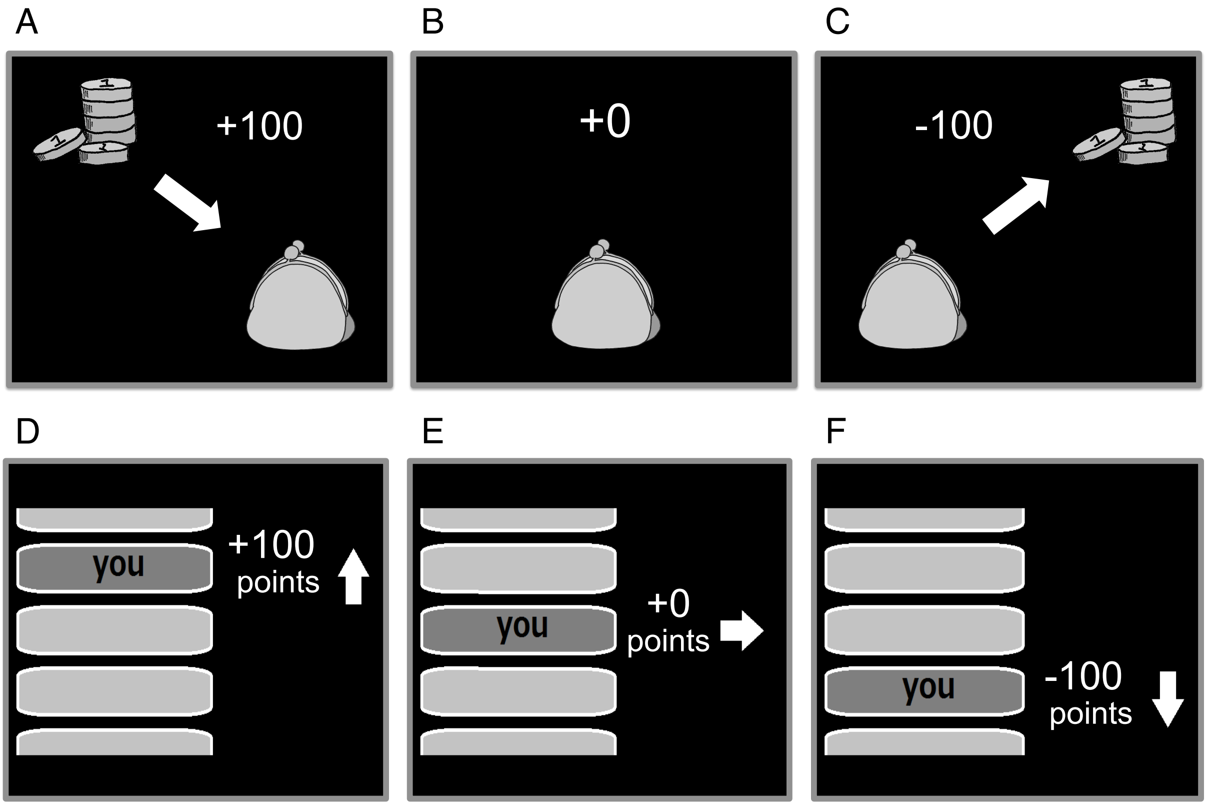

Figure S1. Examples of the feedback illustration of the reward-punishment condition.

[A] and [D] are feedback for 100-points-gained trials in the reward present conditions; [B] and [E] are feedback for the no-gain and no-loss trials in the reward absent conditions; and [C] and [F] are feedback for the 100-points-lost trials in the punishment present conditions. [A], [B], and [C] are for the financial go/no-go task. [D], [E], and [F] are for the non-financial go/no-go task.

Table S1

Mean (SD) of Reaction Time and Error Rate on Each Condition

	Financial feedback				Non-financial feedback			
	PunA		PunP		PunA		PunP	
	RewA	RewP	RewA	RewP	RewA	RewP	RewA	RewP
M_{RT} (ms)	373.2	375.3	370.7	373.9	370.9	375.6	375.4	373.6
	(18.4)	(20.7)	(20.0)	(21.5)	(19.1)	(22.7)	(19.1)	(20.3)
CERT (ms)	364.3	351.6	354.3	360.3	363	342.9	358.1	342.4
	(86.0)	(37.7)	(66.7)	(62.0)	(57.7)	(57.1)	(43.8)	(45.2)
CER (%)	38.6	35.3	35.6	30.9	37.3	33.9	34.5	31.4
	(29.0)	(27.4)	(29.1)	(27.3)	(23.5)	(26.4)	(22.0)	(25.7)
Correct	0.3	0.0	0.4	0.0	0.1	0.0	0.0	0.0
response (*f*)	(1.3)	(0.0)	(2.1)	(0.0)	(0.4)	(0.0)	(0.0)	(0.2)

Note. RewA is reward absent (no-reward), RewP is reward present. PunA is punishment absent (no-punishment), PunP is punishment present. M_{RT} is the mean RT for correct trials: trials with responses of shorter than 150 ms were excluded because it was possible participants had responded before they perceived the stimulus. CERT is the mean RT of CER trials; CER is commission error rate, or a go response for passive stimuli. Correct response (*f*) is the number of correct response shorter than 150ms for active stimuli.

Table S2

The fixed effect of multilevel regression analysis: Commission error RT

					CI			
	β	*SE*	*df*	*t*	2.5%	97.5%	*p*	
(Intercept)	2.699	9.251	128.8	.292	-15.202	21.208	.771	
Age	2.889	2.024	36.0	1.427	-.962	6.747	.162	
Rew	-13.346	7.430	38.3	-1.796	-27.861	1.157	.080	†
Pun	-3.635	6.501	51.0	-.559	-16.164	9.038	.579	
Type	8.641	9.478	31.3	.912	-9.636	27.290	.369	
Order	-.382	1.816	99.0	-.210	-4.073	3.160	.834	
ADHD-RS-IV	.128	.178	36.2	.720	-.214	.471	.476	
ASSQ	-.396	.857	37.4	-.462	-2.070	1.280	.647	
Age X Rew	-8.830	3.420	42.1	-2.582	-15.585	-2.240	.013	*
Age X Pun	-4.368	3.033	56.2	-1.440	-10.265	1.505	.155	
Age X Type	10.426	4.563	37.3	2.285	1.757	19.625	.028	*
Rew X Pun	11.854	11.598	161.9	1.022	-10.202	34.186	.308	
Rew X Type	9.351	11.558	162.6	.809	-12.599	31.612	.420	
Pun X Type	-.483	11.633	164.5	-.042	-22.617	21.931	.967	
Age X Rew X Pun	2.639	5.477	170.3	.482	-7.784	13.227	.631	

Table S2 (continuation)

Age X Rew X Type	-7.772	5.428	171.6	-1.432	-18.104	2.758	.154
Age X Pun X Type	-6.067	5.487	176.3	-1.106	-16.484	4.581	.270
Rew X Pun X Type	17.200	23.151	162.5	.743	-26.857	61.938	.459
Age X Rew X Pun X Type	1.622	10.904	172.4	.149	-19.089	22.991	.882

Note. Rew: Reward; Pun: Punishment; Type: feedback type.

Table S3

The fixed effect of multilevel regression analysis: Commission error rate

					CI			
	β	*SE*	*df*	*t*	2.5%	97.5%	*p*	
(Intercept)	-.583	2.489	102.7	-.234	-5.392	4.190	.815	
Age	-4.968	.813	36.4	-6.115	-6.523	-3.416	.000	***
Rew	-3.191	1.318	121.9	-2.421	-5.726	-.655	.017	*
Pun	-2.222	1.594	38.3	-1.394	-5.339	.896	.171	
Type	-.160	1.843	37.7	-.087	-3.753	3.412	.931	
Order	.130	.384	99.2	.337	-.619	.898	.737	
ADHD-RS-IV	.079	.070	36.1	1.132	-.062	.220	.265	
ASSQ	-.192	.331	36.1	-.581	-.854	.466	.565	
Age X Rew	.348	.590	122.3	.589	-.788	1.483	.557	
Age X Pun	-.062	.713	38.3	-.086	-1.424	1.298	.932	
Age X Type	-1.681	.876	41.9	-1.920	-3.399	.010	.062	†
Rew X Pun	-2.065	2.573	189.1	-.802	-6.899	2.766	.423	
Rew X Type	.201	2.573	189.1	.078	-4.632	5.134	.938	
Pun X Type	-.072	2.576	189.6	-.028	-4.906	4.770	.978	
Age X Rew X Pun	-1.539	1.151	189.1	-1.337	-3.703	.621	.183	

Table S3 (continuation)

Age X Rew X Type	1.664	1.151	189.1	1.446	-.497	3.870	.150
Age X Pun X Type	.590	1.151	189.2	.512	-1.576	2.749	.609
Rew X Pun X Type	-3.645	5.148	189.2	-.708	-13.312	6.024	.480
Age X Rew X Pun X Type	.063	2.303	189.2	.027	-4.261	4.389	.978

Note. Rew: Reward; Pun: Punishment; Type: feedback type.

Table S4

The participants who did not present any incorrect responses

		RewA-PunA	RewP-PunA	RewA-PunP	RewP-PunP
Financial feedback	*N*	7	6	5	9
	*M*age	14.3	13.3	13.8	13.0
	*SD*age	0.8	1.9	1.6	1.7
Non-financial feedback	*N*	2	6	3	7
	*M*age	14.5	13.5	14.0	13.4
	*SD*age	0.7	1.4	1.0	2.1

Note. RewA-PunA: the no reward-no punishment condition; RewP-PunA: the reward condition; RewA-PunP: the punishment condition; RewP-PunP: the reward-punishment condition. Total number of participants was eighteen unlike total because some participants show no incorrect response among some blocks (*Min* 1, *Max* 7, *M* = 2.5, *SD* = 1.8).
